# Dynamic Molecular Simulation of Polyethylene/Organoclay Nanocomposites for Their Physical Properties and Foam Morphology

**DOI:** 10.3390/ma16083122

**Published:** 2023-04-15

**Authors:** Rahida Wati Sharudin, Nik Salwani Md Azmi, Anuaruddin Hanizan, Suffiyana Akhbar, Zakiah Ahmad, Masahiro Ohshima

**Affiliations:** 1 School of Chemical Engineering, College of Engineering, Universiti Teknologi MARA, Shah Alam 40450, Selangor, Malaysia; 2 School of Civil Engineering, College of Engineering, Universiti Teknologi MARA, Shah Alam 40450, Selangor, Malaysia; 3 Department of Chemical Engineering, Kyoto University, Kyoto 615-8510, Japan

**Keywords:** clay, dielectric constant, interfacial tension, molecular modelling, polyethylene

## Abstract

Polyethylene materials are of great interest to be used in many applications due to their many advantageous characteristics. It is light, highly chemical resistant, easy to process, low in cost and has good mechanical properties. Polyethylene is widely used as a cable-insulating material. However, research is still needed to further improve its insulation quality and properties. In this study, an experimental and alternative approach through a dynamic modeling method was conducted. The main objective was to investigate the effect of modified organoclay concentration on the properties of polyethylene/organoclay nanocomposites by observing their characterization and optical and mechanical properties. The thermogram curve reveals that 2 wt% organoclay used has the highest crystallinity (46.7%) while the highest amount of organoclay used produced the lowest crystallinity (31.2%). The presence of cracks was also observed mostly in the nanocomposite with higher content of organoclay, usually where 2.0 wt% and above of organoclay was used. Morphological observation from simulation results supports the experimental work. Only small pores were observed to form in lower concentrations, and as the concentration was increased to 2.0 wt% and above, the pores present became larger in size. Increasing the concentration of organoclay up to 2.0 wt% reduced the interfacial tension while increasing the concentration above 2.0 wt% did not bring any changes to the interfacial tension value. Different formulations produced different behavior of nanocomposite. Hence the control of the formulation was important to control the final result of the products for appropriate application in different sectors of industry.

## 1. Introduction

Polymers are renowned for their insulating properties [1]. In the high-voltage industry, polymers are introduced as the next generation of cable insulation material capable of enhancing the levels of operating voltage and increasing electrical performances [2]. Among the different types of polymers, polyethylene is the most suitable material to be used as insulation material due to its high breakdown strength under electrical stress [3] and low permittivity [2]. Additionally, polyethylene is known as a versatile thermoplastic with good mechanical properties, lightness, easy processing, low cost, chemical resistance, and resistance to abrasion [4]. To date, polyethylene has been widely used in many applications, and it has become the highest volume of plastic sold [5]. Although polyethylene is a good insulation material, there is still a limitation to its properties, such as deterioration of the materials and the maximum operating temperature, which is about 70 °C. This leads to a new solution of crosslinking the polyethylene, which enhances the thermal resistance from 70 °C to 90 °C and the aging stability [6]. In the beginning, the crosslinked polyethylene was manufactured using steam for heating and pressurizing and water for an under-pressure cooling process. During these processes, exposure to a high level of moisture would produce impurities in the cable insulation and lead to the premature breakdown of the insulation. The problem motivates a study on developing a new manufacturing process without involving steam instead of applying electrical heating and pressurizing with dry nitrogen [2]. The failure rate of the crosslinked polyethylene cable has decreased drastically since that time [7].

Crosslinked polyethylene cables are widely used in the present times and are expected to be constantly used in the future [8]. However, the research and development of further increasing the insulation quality continue, and it becomes more difficult to improve the insulation quality mainly because of the cost reduction. This, in turn, encourages the need to explore other alternatives, for instance, the use of polymer-based nanocomposite materials to improve insulation quality [2]. The implementation of polymer-based nanocomposites is largely influenced by the agglomerations of particles because the nanoparticles tend to aggregate together, especially if a polarity difference exists. Nanoparticles also tend to aggregate to minimize the high surface energy [9]. This could be a problem since a polymer with nanoparticles can enhance only its gas-barrier properties if the nanoparticles are properly dispersed in the matrix [10]. Hence, the surface of the nanoparticles is often tailored to maintain aging stability and avoid the aggregation of nanoparticles [11,12]. Nalini et al. [13] showed that a nanocomposite blend prepared from thermoplastic polyolefin with 5% organoclay and 5% compatibilizer could produce an improvement of the mechanical and thermal properties of the nanocomposites due to the polarity differences between the polymer matrix and organoclay surface. The study of the interface is one of the critical aspects in the preparation of the polymer nanocomposites since it is associated with the dispersions of the clay and the interfacial property, which is directly related to the dielectric property of polymer nanocomposites [9].

The effect of modified organoclay on the polyethylene (PE)/organoclay nanocomposites is of great interest. It becomes the objective of this study to investigate the effect of the modified organoclay concentration on nucleation and mechanical properties of the PE/organoclay nanocomposites foams. The potential of using modified organoclay (i.e., Cloisite 10A) as a nucleation agent in the PE matrix was investigated with the intention of controlling its foams. The modification to the organoclay ensures changes to the polarity of the organoclay, which affects the PE matrix and bubble nucleation in its foaming process. The experiments were conducted to investigate the effects of the modified organoclay on the thermal and foaming behavior of PE/organoclay composites by changing the clay concentrations. An alternative method of investigating the interaction between PE and organoclay was also adopted. A recent development in the area of molecular modeling offers the method [14]. Molecular modeling is the method and it was founded on molecular structure through computational chemistry and model building. The computations involve ab initio calculations and quantum mechanics, Monte Carlo, free energy and solvation, and molecular dynamics. Through molecular modeling, a systematic approach is achieved in studying the structural/thermodynamics pattern, testing and developing hypotheses, and interpreting the experimental results [15]. Molecular modeling simulation has been comprehensively used as a process simulator to forecast the expected outcome of certain processes. It is a useful tool that allows more focused testing based on structural and energetic calculations instead of trial and error [16]. Therefore, dynamic molecular modeling was adopted in this study to predict the optical and mechanical properties of PE/organoclay nanocomposites.

## 2. Materials and Methods

### 2.1. Sample Preparation and Foaming Process

The binary nanocomposites of PE and organoclay were prepared by a melt mixing method. The mixing formulation is given in Table 1. The Brabender Plastograph EC Plus (model M-815653) was used for mixing at the rotation speed of 60 rpm, at the temperature of 150 °C, and 10 min of mixing time. In order to avoid any electrical defect of the organoclay caused by the moisture content, the powder of Cloisite 10A was dried in a vacuum oven for 24 h at the temperature of 100 °C. The compounded nanocomposites were then compressed using a hydraulic heat press with a maximum pressure of 200 kg/cm at 180 °C for 5 min. High-pressure compression was utilized in this process to reduce the development of pores caused by the crosslinking by-product in the samples. The samples were cooled down at room temperature. All the samples were degassed in the vacuum oven at 100 °C for 24 h to remove any moisture produced during the compression process. The sample was characterized using differential scanning calorimetry (DSC) to measure the melting and crystallization temperatures as well as the crystallinity of the prepared nanocomposite samples. The measurements were conducted with nitrogen gas purge at a constant flow of 40 mL/min. The heating and cooling rates were set to 10 °C/min in a thermal range from 50 °C to 180 °C for all samples.

The pressure quench foaming method was performed to foam the samples of 2.0 mm in thickness. The sample was placed inside a high-pressure stainless-steel autoclave. The vessel was then heated up and pressurized to 10 MPa using CO_2_ gas. CO_2_ dissolution was conducted at high temperatures of 75 °C, 85 °C, 95 °C, and 105 °C. Finally, the pressure will be quenched to atmosphere pressure within 6 s. The cellular structure of the foams was developed completely during rapid depressurization. The foamed sample was taken out and cooled at room temperature. The foamed PE/organoclay samples were immersed in liquid nitrogen for 10 min and fractured for observation. The cell morphology of the foam was examined using a scanning electron microscope (SEM) at an accelerating voltage of 17 kV.

### 2.2. Molecular Modelling Methods

**Figure 1:**
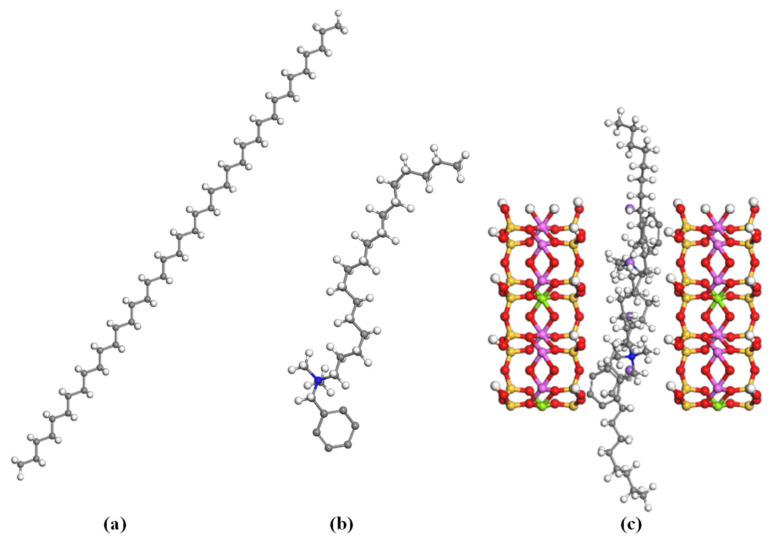
Three-dimensional molecular configuration of (**a**) polyethylene, (**b**) modifier, and (**c**) modified organoclay. (Colour code: red—oxygen; white—hydrogen; blue—ammonium; grey—carbon; yellow—silica; purple—sodium; light purple—aluminum; green—magnesium).

The interaction of polyethylene with modified organoclay was estimated using the molecular modeling simulator Material Studio software. In this work, the polyethylene molecular structure model was constructed using the polymer builder, while CO_2_ and modified organoclay were constructed using the sketching tools package included in the material visualizer. The modified organoclay used in this work was Cloisite 10A with dimethyl-benzyl hydrogenated tallow quaternary ammonium (C18) used as the modifier [17]. The molecular configuration of montmorillonite (MMT) was developed by referring to the work of Scocchi et al. [18]. The basic cell was replicated 2 times in *a* direction and 3 times in a *b* direction with four Al^3+^ ions replaced by the same amount of Mg^2+^. It resulted in the basic unit formula of Na_0.67_[Al_(3.33)_Mg_0.67_]Si_8_O_20_(OH)_4_. The layers are stacked in the *c* direction with a modifier in the middle. The charges were applied to the molecules. Then, all molecular configurations were optimized, and the energy was minimized using a geometry optimization task. The interactions were described by the condensed-phase optimized molecular potential for the atomistic simulation studies (COMPASS) force field [19], where it was able to predict the properties of an extensive range of organic and inorganic materials [20,21,22]. Figure 1 shows the optimized three-dimensional molecular configuration of (a) polyethylene, (b) modifier, and (c) Cloisite 10A, where red signifies oxygen, white is hydrogen, grey is carbon, blue is ammonium, yellow is silica, purple is sodium, light purple is aluminum, and green is magnesium. All molecules are displayed in a ball and stick configuration.

The polyethylene simulation cell was constructed in a 100 × 100 × 60 Å dimension, which contains a fixed amount of 41 molecules of polyethylene. It is noted that the calculation of cells and the number of molecules is based on the calculations from the experimental data. The polymer chain is usually long, and it has to be shorter than the simulation cell to adequately express the motion of the polymer. However, only 20 monomer polyethylene was used in this work. It is established that a 10–20 monomer of polymer is sufficient to represent the polymeric system [23,24,25,26,27] in molecular modeling. If the molecules were too large, it would become a problem to compute and simulate the molecular movement within a reasonable computing time. Moreover, the number of Cloisite 10A was varied according to the weight of the organoclay used. Additionally, 1, 2, 4, 5, and 10 organoclay were used for 0.5, 1.0, 2.0, 2.5, and 5.0 wt% concentration, respectively. Cloisite 10A was added to the simulation cell randomly, and the cell was refined by subjecting it to the geometry optimization task. Afterward, dynamic simulation was conducted for 100 ps using an NPT ensemble to equilibrate the cell and to find the characteristic of the modified polymer matrix.

CO_2_ cell was constructed with the same dimension containing 1000 molecules. Both cells were combined using the layer tool to form the final simulation cell. The cell was refined once again using a geometry optimization task where 2000 maximum iterations were conducted. A smart algorithm was applied with medium quality where the convergence tolerance of 0.001 kcal/mol for energy and 0.005 kcal/mol/Å for force was used. A dynamic simulation was run with an NVT (constant no of molecules, volume, and temperature) ensemble at 348 K and 378 K, where the temperature was controlled using a Berendsen thermostat. Ewald summation method was used for both Van der Waals interactions and electrostatic interactions with 0.001 kcal/mol accuracy and 0.5 Å buffer width. The simulation was run for 100 ps. The simulation time step was set to 1 fs, and the output trajectories were recorded every 1000 steps for data analysis. For analysis purposes, the CO_2_ molecules were deleted to characterize the properties of PE/organoclay nanocomposite.

### 2.3. Analysis of Data

Analysis of data was conducted after the successful simulations. The optical properties data, such as the dielectric constant, refractive index, and conductivity of PE/organoclay nanocomposites, were analyzed and extracted from the simulation results. From the results of the dynamic simulations, the trajectory data were analyzed frame by frame to obtain the pressure tensor to calculate the interfacial tension. The interfacial tension, *γ*, was calculated from the pressure tensor expression (Equation (1)), where *Lz* is the length of the simulation cell in the *z*-direction and *Px*, *Py*, and *Pz* is the diagonal elements of the pressure tensor [28,29,30].

(1) $$\gamma =-\frac{1}{2}\left(\frac{P_{x}+P_{y}}{2}-P_{z}\right)L_{z}$$

The mechanical properties of PE/organoclay nanocomposites were calculated based on the static constraint method [31,32]. The range set was 0.003. The strain was applied in six directions of *xx*, *yy*, *zz*, *yz*, *zx*, and *xy* of the PE/organoclay model. Through this method, the Lame coefficients *λ* and *µ* were obtained and used to calculate the mechanical parameters, Young’s modulus, *E* (Equation (2)), bulk modulus, *B* (Equation (3)), shear modulus, *G* (Equation (4)), and Poisson’s ratio, *υ* (Equation (5)) [31].

(2) $$E=\mu \;\frac{3\lambda +2\mu }{\lambda +\mu }$$

(3) $$B=\lambda +\frac{2}{3}\mu$$

(4) G=μ

(5) $$\upsilon =\frac{1}{2}\;\frac{\lambda }{\lambda +\mu }$$

## 3. Results and Discussions

### 3.1. Thermal Characterization of PE/Organoclay Nanocomposites

**Figure 2:**
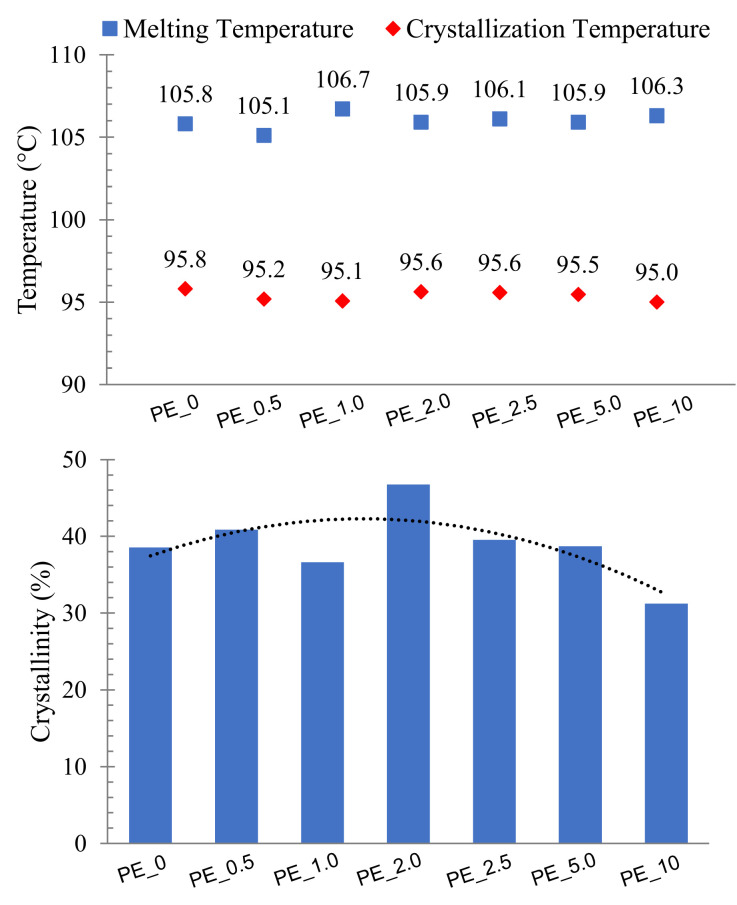
Melting and crystallization temperature and crystallinity of polyethylene nanocomposite produced using different organoclay formulations.

Figure 2 shows the melting and crystallization temperature data extracted from the DSC thermogram curves. The results reveal that there was not much change in both melting and crystallization temperatures of PE nanocomposite with the increase in the concentration of organoclay. The melting temperature of PE nanocomposite was in the range of 105 °C to 107 °C while the crystallization temperature was in the range of 95 °C to 97 °C. Figure 2 also shows the crystallinity of PE nanocomposite formed using a different formulation of organoclay, where the crystallinity of PE nanocomposites relatively increased to a maximum level before it decreased with the increase in the concentration of organoclay in the nanocomposite. PE_2.0 showed the highest crystallinity, 46.7%, compared to the other nanocomposites, while PE_10, which contained the highest amount of organoclay, showed the lowest crystallinity, 31.2%. The crystallinity of the nanocomposite influences the diffusion of CO_2_ and the cellular structure of their foams. Usually, small gas molecules can easily diffuse through the bulk of an amorphous polymer matrix. However, the presence of the crystalline regions hinders this diffusion process by creating a constricted pathway for the diffusing gas molecules [33]. This would also directly affect the final cell structure of the produced foams, especially the uniformity of the cell structure.

### 3.2. Foaming Behaviour of PE/Organoclay Nanocomposites

**Figure 3:**
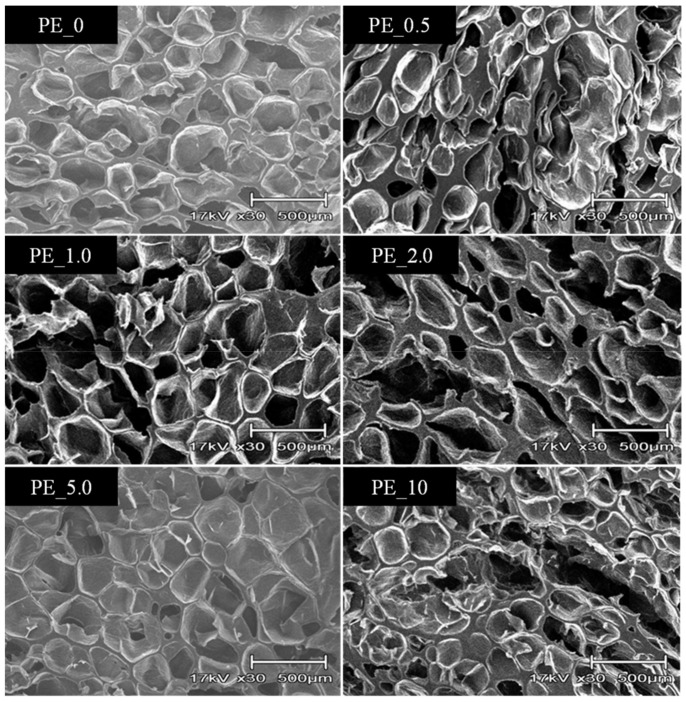
Cellular structure of PE foam produced using different formulations of organoclay at temperature of 105 °C.

**Figure 4:**
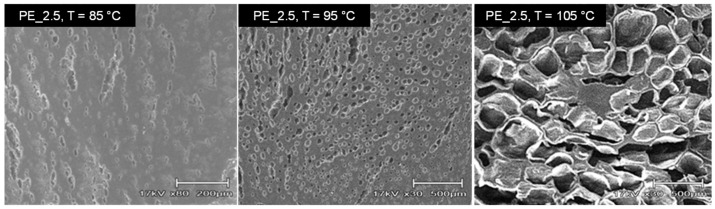
Cellular structure of PE_2.5 foams produced at three temperatures of 85 °C, 95 °C, and 105 °C.

As shown in Figure 3, the nanocomposite produced foams with slightly different cellular structures at the constant temperature of 105 °C. The SEM image shows that PE_2.0 with high crystallinity did not produce a uniform pore size. Although PE_10 has the lowest crystallinity, the cellular structure of its foams was also not uniform, where cracks were observed. The cracks were observed mostly in the PE nanocomposite with the higher contents of organoclay, usually with content over 2.0 wt%. Temperature also played an important role in the foaming process. Figure 4 shows a cellular structure of PE_2.5 foamed at three different temperatures, i.e., 85 °C, 95 °C, and 105 °C. At the foaming temperature of 75 °C, there were fewer visible cells that could be seen in the SEM images, and at 85 °C, the small cell sizes were seen in the SEM image of the foam. The cell size became larger as the foaming temperature approached the nanocomposite’s melting temperature. This is due to the decrease in the viscosity of the PE. The softer region of the PE allows more supercritical CO_2_ to diffuse into the growing bubbles rapidly. Higher diffusion of CO_2_ will increase the cell growth rate, and consequently, the bubble grows faster, creating a larger cell size.

### 3.3. Simulation Calculation of PE/Organoclay Nanocomposite’s Properties

**Figure 5:**
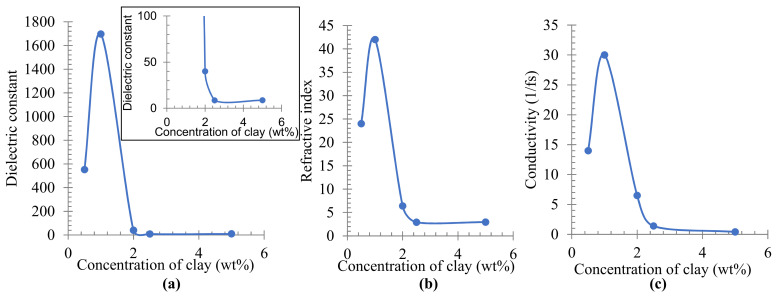
Analysis of (**a**) dielectric constant, (**b**) refractive index, and (**c**) conductivity from molecular dynamic of PE/organoclay nanocomposite, showing a stagnant curve above 2.5 wt%.

The optical properties of PE/organoclay nanocomposites were investigated by molecular modeling technique. Figure 5 shows the dielectric constant, refractive index, and conductivity of PE/organoclay nanocomposites calculated by dynamic simulation. The information on the optical properties of any materials is of great significance for the probable usages as the semiconductor parts and photo-electronic devices. It allows us to understand important features of the materials, such as the electronic structure, the transmission of radiation, and the material’s linear response. The dielectric constant (Figure 5a) can be obtained from the real dielectric function calculated from the imaginary part of the dielectric function [34]. The dielectric constant is a state point-dependent property of a material, mitigating the strength of Columbic interactions, which otherwise would be experienced in a vacuum [35]. The result in Figure 5a shows that the dielectric constant increased as the concentration of organoclay increased from 0.5 to 1.0 wt% and decreased from 1.0 to 2.5 wt% before reaching a constant value in the range from 2.5 to 5.0 wt% of organoclay. A similar trend was also observed at the calculated refractive index and conductivity, which showed the highest value when 1.0 wt% of organoclay was used. Polyethylene is a nonpolar [36] thermoplastic polymer with a long hydrocarbon chain [3]. Despite being nonpolar, the polymer can be made polar by introducing a small amount of additive. In this study, Cloisite 10A was added to the polymer. This, in turn, increased the polarity of the polymer because Cloisite 10A is a polar material [36,37]. An increase in the constant dielectric leads to an increase in electrical conductivity. This explains the increase in dielectric constant and conductivity value illustrated in Figure 5a,c where 0.5 and 1.0 wt% of organoclay was added to polyethylene. As the contents of the organoclay became higher than 2.0 wt%, the dispersion and the distance between the molecules increased. At a closer distance, the interaction and the ability of the molecules to hold the electron cloud and charges became higher, which resulted in a low polarisability and low dielectric constant [36].

The decrease and stagnant curve in the dielectric constant, conductivity, and refractive index with the increase in the amount of organoclay show that PE/organoclay at the different mixing ratios could lead to different applications in many sectors. Material with a higher value of the refractive index can be applied to the field of optical filters, lenses, reflectors, anti-reflection films, and encapsulation materials for optical waveguides [34]. Both low and high dielectric constant values are equally essential in electronic industries [36]. For the application of AC cables and high voltage DC insulating materials, for example, the material must have a lower conductivity than other properties such as low loss factor and absence of electric and electrochemical treeing [2]. Moreover, higher value in dielectric constant and conductivity shows that the polymer nanocomposites have a high potential for use in energy storage application.

**Figure 6:**
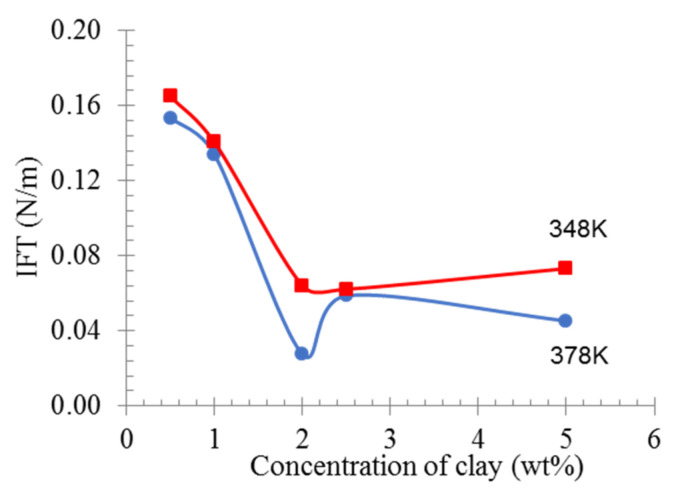
Calculated interfacial tension, IFT, from molecular dynamic of PE/organoclay. The calculated interfacial tension at 248 K is shown in red and interfacial tension at 378 K is shows in blue.

**Figure 7:**
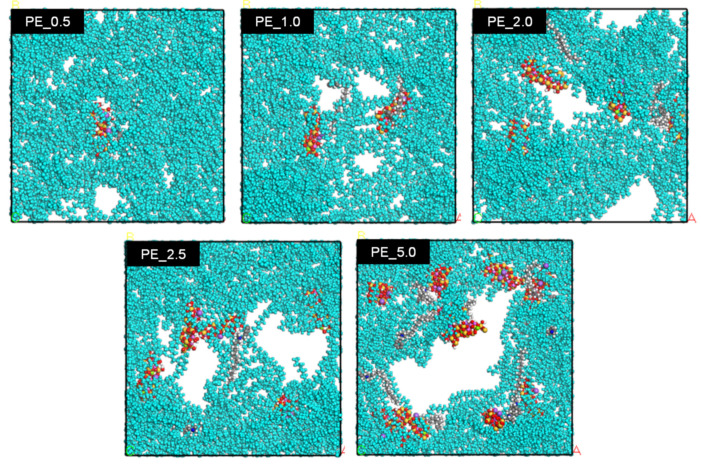
Molecular configuration of PE/organoclay after 100 ps dynamic simulation at 378 K. Colour code: turquois—PE; red—oxygen; yellow—silicon; grey—carbon; white—hydrogen; dark blue—nitrogen; purple —sodium; green—magnesium; pink—aluminum.

Both modifications of nanomaterial and/or polymer matrix are conducted to improve the interfacial compatibility between polymers and nanomaterial, which leads to improved dispersion and results in better mechanical, thermal, and electrical properties. Figure 6 shows the calculated interfacial tension value of PE nanocomposite in different concentrations of organoclay at 348 K and 378 K. CO_2_ molecules were omitted for better observation. The interfacial tension decreased at the range from 0.5 to 2.0 wt% of organoclay content and became constant at the range from 2.0 to 5.0 wt%. It can be considered that the presence of organoclay disturbs the polymer-polymer interaction. It is intended for the nanocomposites to have a lower interfacial tension. In this case, the addition of the organoclay up to 2.0 wt% reduces the IFT with accompanying the change in blend morphology. Increasing the temperature could slightly reduce the IFT, as shown in Figure 6. It was expected that the IFT would keep decreasing with the increase in organoclay content. However, additional organoclay over 2.0 wt% did not change much in the IFT and reached a constant value. The modifier in the organoclay plays an important part in reducing the interfacial tension. The modifier alters the polarity of the organoclay surface and makes the organoclay to be more organophilic [38]. It was achieved by reducing the hydrophilicity and exchanging the polarity of montmorillonite with cation through alkylammonium ions. Even though the organoclay is still a polar material, the alkyl tails of the modifier are hydrophobic. In this study, the hydrophobic alkyl tail facilitates the interaction of the polyethylene chain, reduces the interfacial tension, and helps exfoliate the organoclay. The polyethylene chain, which is also nonpolar and hence hydrophobic, interacts with the alkyl chain and exfoliates the montmorillonite layer. This phenomenon was clearly shown in Figure 7, where all systems showed that the layers of montmorillonite in organoclay were separated as they were filled with polyethylene chains. The modifier also prevents the montmorillonite platelets from stacking together [38,39] due to the steric effect, where they repel each other [38].

**Figure 8:**
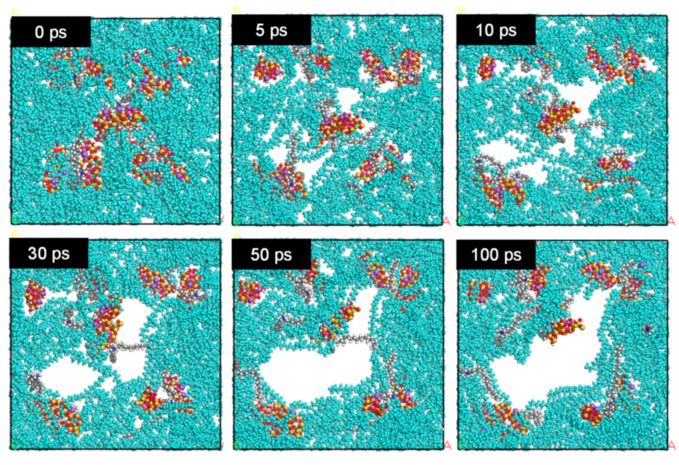
Time evolution of molecular configuration of PE_5.0 at 378 K. Colour code: turquois—PE; red—oxygen; yellow—silicon; grey—carbon; white—hydrogen; dark blue—nitrogen; purple—sodium; green: magnesium; pink—aluminum.

Figure 7 shows the molecular configuration of PE/organoclay systems at different organoclay concentrations at 378 K after 100 ps from the start of the dynamic simulation. For easier observation, the PE molecules were colored in turquoise while other molecules were in their original colors: red is oxygen, yellow is silicone, grey is carbon, white is hydrogen, dark blue is nitrogen, purple is sodium, green is magnesium, and pink is aluminum. When the polymer is subjected to mixing, the particles’ interaction causes agglomeration. The final pore size is determined by the competition between coalescence and break up. As shown in Figure 7, in the presence of 0.5 wt% of organoclay, only small pores were observed. When the content of organoclay increased to 1.0 wt%, more pores were formed. When the organoclay content was further increased to above 2.0 wt%, the pores became larger in size. The molecular configurations of the PE_5.0 at different time frames are shown in Figure 8, which shows a pore formation with the increase in simulation time.

At 0 ps, there is no pore formation observed. The molecules were closely packed together in the simulation cell. Once the dynamic simulation started, the molecules became mobile and moved from their original positions. At 5 ps, pore formation started due to the interaction between molecules. At 10 ps, the pore formation became more prominent. Small pores were observed to form in the early stages of dynamic simulation, and the pore size became larger as the dynamic simulation time elapsed. Eventually, those small pores coalesced together to form one large pore, as shown in the image of 100 ps. These pores would be the origin of the cracks (Figure 3) in the foams of the PE nanocomposite.

**Figure 9:**
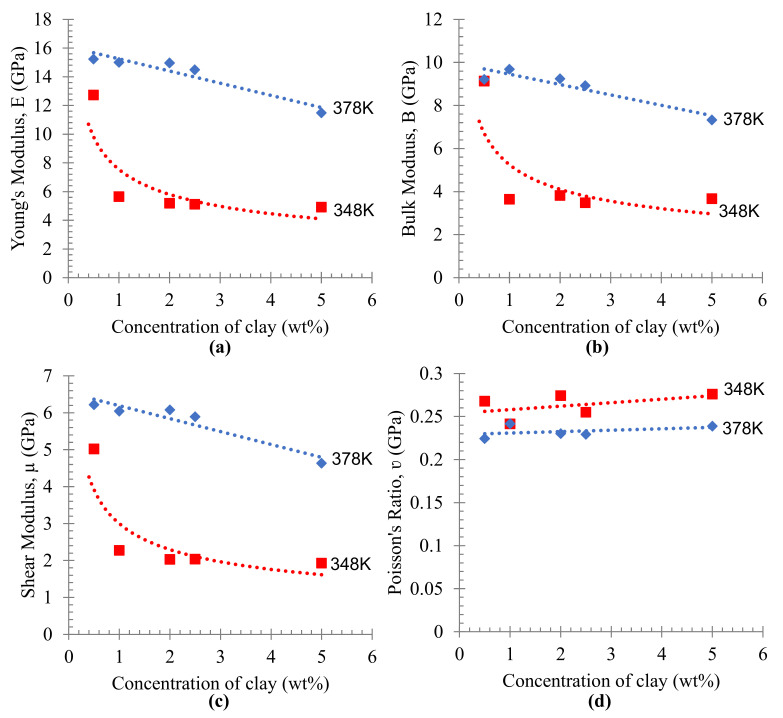
The predicted mechanical properties (**a**) Young’s modulus, (**b**) bulk modulus, (**c**) shear modulus and (**d**) Poisson’s ratio of PE/organoclay produced at different temperatures. The mechanical properties predicted at 348 K and 378 K is shown in red and blue line respectively.

The mechanical properties of PE/organoclay nanocomposite were calculated from the Lame coefficients obtained from a successful dynamic simulation (Figure 9). Young’s modulus (Figure 9a) was used to characterize the rigidity of materials or the affinity of an object to deform under tension or compression [40], bulk modulus *B* (Figure 9b) was used to characterize material incompressibility, shear modulus *µ* (Figure 9c) was used to characterize material’s ability to resist shear strain and Poisson’s ratio, and *λ* (Figure 9d) was used to explain the material plasticity [31]. Young’s modulus (Figure 9a) of the PE/organoclay foam decreased as the content of organoclay increased; however, the PE foam prepared at higher temperatures showed a higher value of Young’s modulus. This shows that the mechanical properties could be improved to some extent by preparing the composites under higher temperatures. The higher modulus materials usually give a little stretch, while the lower modulus materials, such as rubbers and thermoplastics, are stretchy [41]. The bulk modulus (Figure 9b) was decreased slightly when a higher amount of organoclay was used. The materials with higher bulk modulus showed their ability to resist a higher compression load. A similar trend was observed in the shear modulus. It was observed that the ability to resist the shear strain decreased with the increase in the organoclay content (Figure 9c). Moreover, Poisson’s ratio showed no significant changes in the plasticity behavior of PE/organoclay foams against the increase in the organoclay content. However, as the temperature increased, the Poisson’s ratio showed a lower value compared to the values at the lower temperatures (Figure 9d).

### 3.4. Discussion

**Figure 10:**
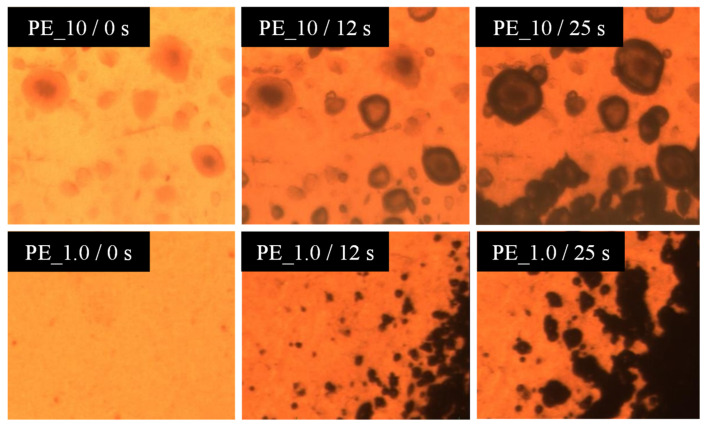
Visual observation of foaming of PE_10 and PE_1.0 in a depressurization process at 105 °C.

The formation of the cracks in the foamed samples is due to the agglomeration of molecules around the organoclay. Figure 10 shows the visual observation of pressure quench foaming of the PE_10 and PE_1.0 samples at 105 °C. Before depressurization at 0 s, there was a black spot on the PE_10 nanocomposites, which corresponded to a clump of organoclay, while PE_1.0 showed no black spot. As the depressurization started for foaming, the formation of bubbles was observed around the black spots, and their size increased as time went out. This phenomenon reaffirms the hypothesis that the organoclay (Closite 10A) acts like a bubble nucleating agent. As the organoclay initiates the bubble nucleation, highly condensed bubbles appear in this region and thus cause major cracks in the foamed samples. Higher agglomeration increased the number of nucleated bubbles and led to coalescence subsequently. As a result, the size of the cracks became larger. Higher organoclay content and higher dispersiveness of organoclay are important to increase the number of cells. However, too much organoclay content may bring an undesired effect on the foam morphology. Dynamic simulation supports the hypothesis that the high amount of organoclay content produces larger size pores in foams (Figure 7). The dynamic simulation elucidated that the pores overlapped as the dynamic time progressed, and eventually, they were combined to form a larger pore (Figure 8). Adding the organoclay to the PE disturbs the polymer–polymer interaction and consequently reduces the interfacial tension. By the selective localization of the organoclay at the interface and its distribution in the polymer, the coalescence between particles could be suppressed [42]. Thus, it can be said that the alteration of the polarity at the organoclay surface successfully reduces the interfacial tension and enhances the compatibility between the organoclay and polyethylene chain. This prevents the montmorillonite layers from sticking together. By the exfoliation of the montmorillonite layer, interaction and localization of organoclay particles successfully occur at the interface. From the viewpoint of the dielectric materials application, this interfacial region may serve as a trapping site for the charge carried by the polar nanoparticles. As the density of trapping sites increases, carrier mobility and energy are reduced. By reducing the energy at the concerned region, the materials may have less damage and may extend their lifetime. It is presumed that this phenomenon can be well explained by the dynamic simulation where the trend for dielectric constant and conductivity reduced with the decrease in the interfacial tension at higher content of organoclay used in the PE matrix.

## 4. Conclusions

PE nanocomposites with different contents of organoclay showed the different crystallinity and cellular structure of their foams. The increase in the organoclay up to 2.0 wt% increased the crystallinity; however, the further increase in the clay content decreased the crystallinity of polyethylene. Higher crystallinity hindered the diffusion of the physical blowing agent, CO_2_, during the foaming process by restricting the diffusion pathway of CO_2_ molecules in the polymer. Although it was expected that the composite with the lowest crystallinity produced a uniform cellular structure of its foams, it is not always the case, as different observation was recorded for the foamed PE_10. PE_10 had the lowest crystallinity, but the cellular structure of its foams was not uniform. Instead, cracks were formed due to the overlapping of pores produced because the concentration of organoclay used was too high. By employing the molecular dynamic simulation, the interaction of polyethylene and the modified organoclay and pore formation in compounds were successfully simulated. The optical properties of PE/organoclay nanocomposite show the potential of the nanocomposite for having several applications in different sectors, and these properties predicted by the molecular simulation would be useful in determining its application in industry. The dielectric constant, refractive index, and conductivity value showed the lowest value as a higher amount of organoclay was used. The lower dielectric constant and conductivity are crucial indicators for the application of AC cables and high-voltage DC insulations. The predicted mechanical properties also show that an improvement can be achieved to a certain extent by preparing the materials at higher temperatures.

## Figures and Tables

**Table 1:** PE/organoclay nanocomposites composition.

Sample	Organoclay (wt%)
PE_0	0.0
PE_0.5	0.5
PE_1.0	1.0
PE_2.0	2.0
PE_2.5	2.5
PE_5.0	5.0
PE_10	10.0

## Data Availability

Data sharing not applicable.
